# Transcriptomic and phenotype analysis revealed the role of *rpoS* in stress resistance and virulence of pathogenic *Enterobacter cloacae* from *Macrobrachium rosenbergii*

**DOI:** 10.3389/fmicb.2022.1030955

**Published:** 2022-11-10

**Authors:** Xiaojian Gao, Qieqi Qian, Yujie Zhu, Zhen Chen, Jingwen Xu, Wenjing Xu, Qun Jiang, Jun Wang, Xiaojun Zhang

**Affiliations:** College of Animal Science and Technology, Yangzhou University, Yangzhou, China

**Keywords:** *Enterobacter cloacae*, *rpoS*, transcriptomic analysis, stress resistance, virulence

## Abstract

*Enterobacter cloacae* is widely distributed in the aquatic environment, and has been determined as a novel pathogen of various aquatic animals recently. Our previous studies have indicated *E. cloacae* caused repeated infections in *Macrobrachium rosenbergii*, suggesting a high survival ability of the bacteria, and *rpoS* gene has been known to regulate stress response and virulence of many bacteria. In this study, the *E. cloacae*-*rpoS* RNAi strain was constructed by RNAi technology, and the regulation role of *rpoS* in stress resistance and virulence of *E. cloacae* was explored by transcriptomic and phenotype analysis. The transcriptome analysis showed a total of 488 differentially expressed genes (DEGs) were identified between *rpoS*-RNAi and wild-type strains, including 30 up-regulated genes and 458 down-regulated genes, and these down-regulated DEGs were mainly related to environmental response, biofilm formation, bacterial type II secretory system, flagellin, fimbrillin, and chemotactic protein which associated with bacterial survival and virulence. The phenotype changes also showed the *E. cloacae*-*rpoS* RNAi strain exhibited significantly decreasing abilities of survival in environmental stresses (starvation, salinity, low pH, and oxidative stress), biofilm production, movement, adhesion to cells, pathogenicity, and colonization to *M. rosenbergii.* These results reveal that *rpoS* plays an important regulatory role in environmental stress adaptation and virulence of *E. cloacae.*

## Introduction

*Enterobacter cloacae* an enteric bacterium that belongs to *Enterobacteriaceae*, is widely distributed in various aquatic and terrestrial environments (Krzymińska et al., 2010), and has been considered as an opportunist pathogen causing variety of infections both in human and animals (Lee et al., 2017). Furthermore, the control of *E. cloacae* mainly relies on antibiotics, which leads the emergence of multi-resistant *E. cloacae* strains (Band et al., 2016). Recently, *E. cloacae* has been recognized as an aquatic pathogen to several aquatic animals, e.g., *Macrobrachium rosenbergii* (Gao et al., 2019, 2020, 2021), *Procambarus clarkia* (Dong et al., 2020), *Mugil cephalus* (Thillai et al., 2008), and *Coryphaena hippurus* (Hansen et al., 1990). Our previous studies also showed the *E. cloacae* caused repeated infections in *M. rosenbergii* (Gao et al., 2019, 2021), suggested that *E. cloacae* has developed survival strategies to adapt various stresses in the aquatic environment. Pathogenic bacteria of aquatic animals usually encounter various stresses in the aquatic and host environments, such as nutrient starvation, oxidative stress, changes in pH, temperature and osmolality, etc. Hence, the bacteria overcome these environmental stresses require self-regulation of major genes expression that commonly controlled by sigma factors (Qiu et al., 2013).

Sigma factors are considered as important regulators involved in transcription and regulation of various genes, which regulate many cellular activities, such as growth, environmental stresses adaptation, biofilm formation, and virulence (Feng et al., 2021). Among these, the alternative sigma factor (RpoS) has been widely considered as a key regulatory factor, which is responsible for regulating stress response in many bacteria (Landini et al., 2014). Previous studies showed that RpoS plays important roles in tolerance of high temperature, salt stress, acid stress and many other environmental stresses, e.g., the *rpoS* mutant strains of the bacteria such as *Vibrio alginolyticus*, *Edwardsiella tarda*, *Escherichia coli*, *Shewanella baltica*, *Serratia plymuthica*, and *Yersinia pseudotuberculosis* exhibited significantly decreasing abilities of survival in various stresses compared to the wild type (Battesti et al., 2011; Liu et al., 2014, 2016; Guan et al., 2015; Huang et al., 2019; Zhang et al., 2021).

In addition to its role in the stress response, RpoS also plays important regulatory roles in virulence of pathogenic bacteria (Yin et al., 2018). RpoS is responsible for regulating virulence by increasing resistance to host environment stresses and regulating expression of virulence-related genes in serval bacteria (Dong and Schellhorn, 2010). Hülsmann et al. (2003) reported that RpoS could positively regulate many extracellular enzymes activities, which may be required for invasion to host. Similarly, Ma et al. (2009) reported that the *rpoS* mutant of *Vibrio anguillarum* decreased markedly production of extracellular enzymes and virulence to zebra fish. RpoS also controls the expression of various virulence genes, e.g., RpoS could positively regulate the expression of *spvR* and *spvABCD* virulence genes, which are responsible for causing disease in mice and humans (Fang et al., 1992; Norel et al., 1992).

Although RpoS has been recognized as an important regulator in stress resistance and virulence of many bacteria, its regulatory mechanism in *E. cloacae* is still scarce. In this study, the knockdown of *rpoS* in *E. cloacae* was constructed by RNA interference (RNAi), growth curves, stress response, biofilm formation, adhesion ability, virulence, and transcriptomic changes between *rpoS*-RNAi and wild strains, were investigated. Our results will contribute to reveal the regulatory mechanisms of *rpoS* in stress response and virulence of *E. cloacae*, and provide theoretical support for prevention and control the diseases caused by *E. cloacae* in aquatic animals.

## Materials and methods

### Bacterial strains and growth conditions

The *E. cloacae* XL3-1 strain was isolated from the diseased *M. rosenbergii* in Gaoyou County of Jiangsu Province (Gao et al., 2019), and incubated in LB medium at 28°C with shaking at 180 rpm for 18 h. *E. coli* DH5α (TransGen Biotech, Beijing, China) incubated in LB medium at 37°C with shaking at 180 rpm for 18 h.

### Stable gene silence

The *E. cloacae*-*rpoS* RNAi strain was performed following previously described methods (Luo et al., 2019). Five short hairpin RNA sequences, respectively, targeting the *rpoS* (Genbank accession number: Y13230.1) were designed by Invitrogrn Block-iT RNAi Designer and synthetized by Shanghai Generay Biotech Co., Ltd. (Shanghai China). The designed synthetic shRNA was annealed, and then ligated to the pCM130/tac plasmid after digestion with the enzymes *Nsi*I and *Bsr*GI (New England Biolabs) to construct pCM130/tac-*rpoS*. The recombinant plasmid was transformed into *E. coli* DH5α by heat shock, and then extracted and introduced into *E. cloacae* by electroporation. The *rpoS*-RNAi *E. cloacae* was screened by tetracycline (10 μg/mL) and qRT-PCR.

### Transcriptome sequencing and data analysis

Total RNA was extracted from the wild-type and *rpoS*-RNAi *E. cloacae* (*n* = 3) for cDNA preparations, and the contaminating genomic DNA was removed by RNase-free DNase I. Further, the RNA quantity and quality were assessed using Agilent 2,100 Bioanalyzer (Agilent technologies, United States) and NanoDrop 2000 (Thermo Fisher Scientific, United States). Then, rRNA from the total RNA of each sample was removed, followed by RNA fragmentation. The first strand cDNA was synthesized with random primers and M-MuLV reverse transcriptase. Second strand cDNA synthesis was subsequently performed using DNA Polymerase I and RNase H. Prior to final amplification, the synthesized cDNA fragments were purified, end repaired, ligated to sequencing adapters, and selectively degraded with USER enzyme. After that, sequencing was carried out on the Hiseq2000 (Beijing Novogene Co., Ltd., Beijing China). Then the clean reads were obtained from raw reads by removing adapters, higher N rate reads, and low quality reads. Then the sequencing reads were compared with the reference genome (GenBank accession number: CP012162.1) using Bowtie software. The gene expression levels were calculated by using FPKM (Fragments Per Kilobase of transcript per Million mapped reads) method. The criteria of differentially expressed genes (DEGs) was log2(FoldChange)| > 0 and padj <0.05. The enrichment analysis of DEGs were conducted by using Gene Ontology (GO) and Kyoto Encyclopedia of Genes and Genomes (KEGG) enrichment analysis (Conesa et al., 2005; Kanehisa et al., 2014).

### Quantitative real-time PCR analysis

The qRT-PCR was carried out using Thermofisher QuantStudio Real-Time PCR System PCR System with ChamQ Universal SYBR qPCR Master Mix (Vazyme, Nanjing Co., Ltd., China), and the primer sequences are displayed in Supplementary Table S1. The 16S rRNA was chosen as an internal control, and relative mRNA expression was calculated by the 2^−ΔΔCt^ method.

### Growth assay

Overnight cultures of wild-type and *rpoS*-RNAi *E. cloacae* were adjusted to OD_600_ = 0.5, diluted 1:100 into LB medium, and then incubated at 28 h with shaking for 24 h. The values of OD_600_ were recorded at 2-h intervals, the growth curves were conducted both the wild-type and *rpoS*-RNAi strains.

### Motility assay

The concentration of overnight cultured wild-type and *rpoS*-RNAi *E. cloacae* was adjusted to OD_600_ = 0.3. 1 μl of the bacterial suspension was spotted into LB medium with 0.4% agar, incubated at 28°C for 24 h, and then the diameters of the colonies surrounding the punctured portion of the agar media were measured (Wang et al., 2019).

### Biofilm formation

The biofilm formation assay for *E. cloacae* was conducted as described by Huang et al. (2019). The overnight cultures of wild-type and *rpoS*-RNAi *E. cloacae* were adjusted OD_600_ = 0.2. Then 100 μl of bacterial suspension was added to a 96-well plate and incubated at 28°C for 24 h. After incubation, the plate was rinsed three times with sterile PBS (pH 7.4), stained with 200 μl 1% crystal violet for 15 min, and then the plate was rinsed again and air-dried. Finally, 200 μl of 95% ethanol solution was added to solubilize the stained biofilm, which was measured at OD_590_ nm using a microplate reader. The above results were normalized by the bacterial numbers and conducted for six replicates.

### Adhesion assays

Overnight cultures of wild-type and *rpoS*-RNAi *E. cloacae* were adjusted to OD_600_ = 1, the bacteria were 10-fold serially diluted using sterilized PBS (pH 7.4), and then then bacterial colonies were counted by plating 100 μl of bacteria solution on LB agar. For adhesion assay, the human renal epithelial cells (293 T) were subsequently infected at a multiplicity of infection (MOI) of 10 for 2 h. The infected cells were rinsed with PBS three times to remove non-adherent bacteria. Adherent bacteria were isolated from 293 T cells using 0.1% Triton-X 100. The serial dilution of collected bacterial suspension was coated onto LB plates, and then bacterial colonies were counted. The percentage of adhesion was calculated as adherent CFU × 100/total inoculum CFU. The assay was performed in triplicate.

### Stress survival assay

The wild-type and *rpoS*-RNAi *E. cloacae* were grown in LB medium at 28°C to stationary phase. For osmotic stress assay, the cultures were diluted 50-fold into containing 4% NaCl. For acid stress assay, the cultures were diluted 50-fold into LB medium which were adjusted to pH 4.0. For H_2_O_2_ stress assay, the cultures were diluted 50-fold into LB medium with 1.5 mM H_2_O_2_. In addition, the cultures of wild-type and *rpoS*-RNAi *E. cloacae* were also diluted into normal LB medium as control groups. After incubating at 28°C for 1 h, the cultures were serially diluted and plated onto LB agar plates, colonies were counted after 24 h incubation. Additionally, the starvation stress assay was performed as described by Gao et al. (2022), the starved cells were sampled at day 7, 14, 21, and 28 for colonies counts. The survival rate was calculated as follows: (CFU of stressed cells/CFU of untreated cells) × 100%. The assay was performed in triplicate.

### Virulence test

To investigate pathogenicity of wild-type and *rpoS*-RNAi *E. cloacae*, the 2 months after hatching old *M. rosenbergii* (0.23 ± 0.05 g) were obtained from a local farm in Gaoyou county and acclimated for a week. These prawns were divided into test and control groups, the test groups were infected by exposure to 2.4 × 10^7^ CFU/mL of wild-type and *rpoS*-RNAi *E. cloacae*, respectively, while the control group prawns were cultured in fresh water without any bacteria inoculation. The experimental *M. rosenbergii* were kept at 28 ± 2°C, monitored for 96 h without feeding and water change. The mortalities of prawns were monitored at 6, 12, 24, 48, 72 and 96 h, the dead *M. rosenbergii* were immediately removed and re-isolated to confirm the mortality caused by *E. cloacae*. In addition, the bacterial burden in the prawns was detected at 12, 24, and 48 h. 100 mg of prawns was homogenized in 1 ml of sterile PBS (pH 7.4), and the serial diluted homogenates were plated onto LB agar plates for bacterial colonies counting.

### Statistical analysis

Statistically significant differences among wild-type and *rpoS*-RNAi *E. cloacae* groups were analyzed one-way ANOVA using SPSS 16.0 software. The results data were expressed as means ± standard deviation (SD), and *p* < 0.05 was considered as significant difference.

## Results

### Construction of the *rpoS*-RNAi strain

**Figure 1:**
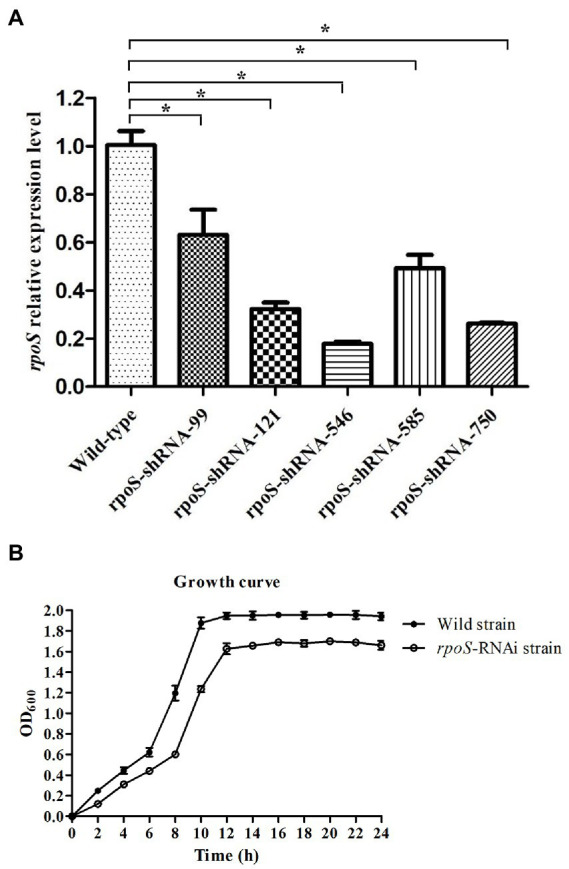
Construction of RNAi strains of *Enterobacter cloacae*. **(A)** Gene expression levels of *rpoS* after stable gene silencing, the asterisk indicated significant differences between the RNAi and wild *E. cloacae*, *p* < 0.05. **(B)** Growth curve of wild-type and *rpoS*-RNAi strains.

The expression of *rpoS* in the wild-type and rpoS-RNAi E. cloacae was compared. All of the five shRNAs significantly decreased the expression of *rpoS* with different efficiency, and the reduction in the expression level of *rpoS*-shRNA-99, *rpoS*-shRNA-121, *rpoS*-shRNA-546, *rpoS*-shRNA-585 and *rpoS*-shRNA-750 was 37.30, 68.06, 82.62, 51.09, and 74.21%, respectively (Figure 1A). Therefore, the *rpoS*-shRNA-546 was selected for further studies. In addition, the growth curves showed that the growth rate of *rpoS* silenced strain was lower than that of the wild-type (Figure 1B).

### Transcriptome changes induced by knockdown of *rpoS*

**Figure 2:**
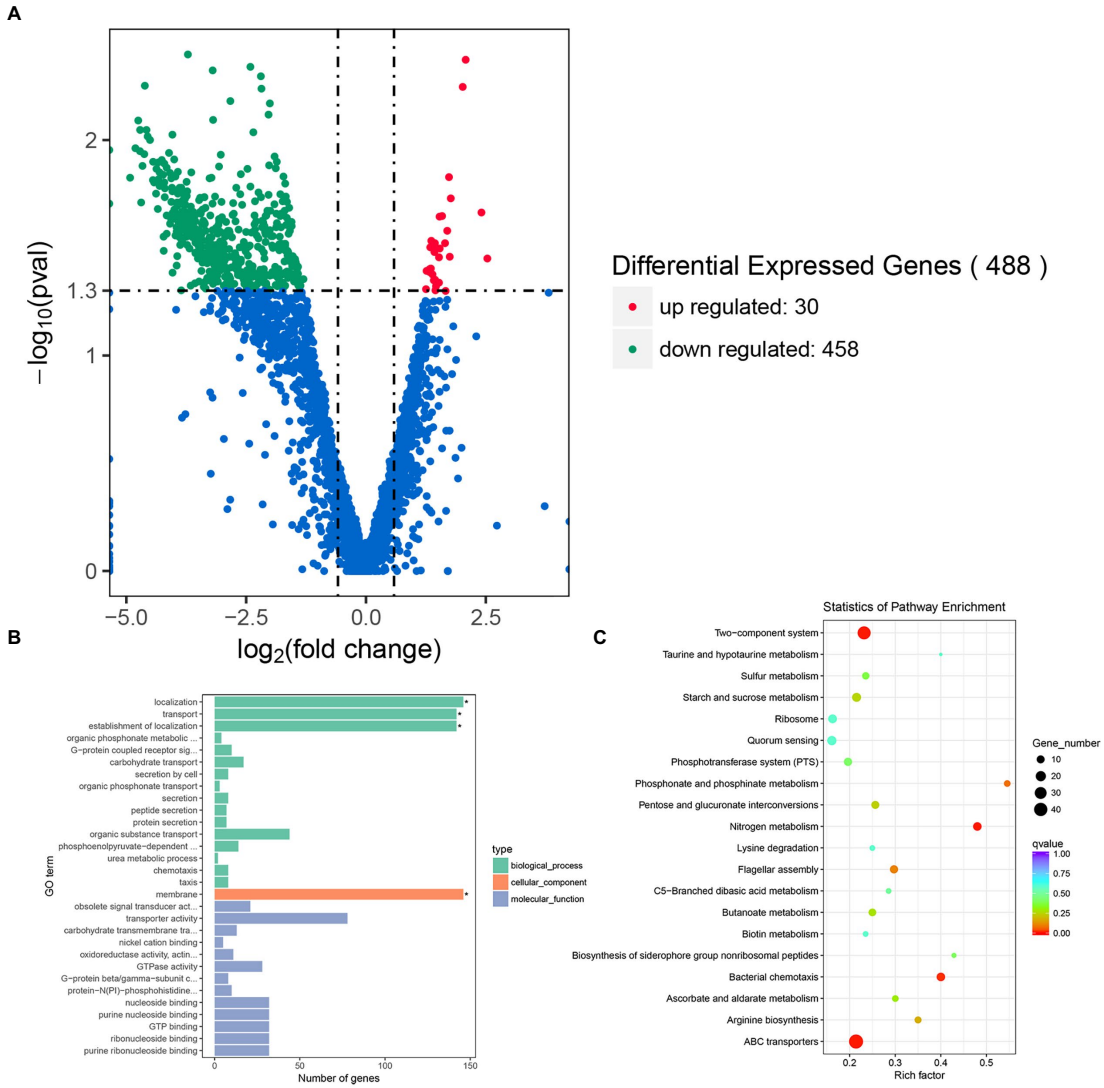
Transcriptomic analysis of *E. cloacae* between *rpoS*-RNAi and wild-type strains. **(A)** The volcano plot of DEGs, the red dots were up-regulated genes and the green dots were down-regulated genes. **(B)** GO analysis of differential expression genes of *rpoS*-RNAi and wild-type strains. **(C)** The Top20 KEGG enrichment pathways for DEGs, the rich factor refers to the ratio of the number of DEGs in the pathway and the number of all annotated genes in the pathway.

To investigate the regulatory role of *rpoS* in *E. cloacae*, transcriptome profiles of the *rpoS*-RNAi and wild-type *E. cloacae* were analyzed using RNA sequencing. After filtering through the raw reads, a total of 11,583,012, 10,789,068, 10,939,872 clean reads from *rpoS*-RNAi strains, 11,649,402, 9,878,616, 14,998,822 clean reads from wild-type strains were obtained, respectively (Supplementary Table S2). A total of 488 DEGs in *rpoS*-RNAi were identified in comparison with the wild-type strain, including 458 downregulated genes and 30 upregulated genes (Figure 2A). Among DEGs, genes responsible for resistance to environmental stress (e.g., *bfr*, *dps*, *funC*, *katE*、*uspB*, *uspC*, *uspE*, and *uspF*), flagellar assembly (*flgC*, *flgF*, *fliF*, *fliG*, *fliH*, *fliI*, *fliK*, *fliO*, *flhA*, *flhB*, and *fimC*), fimbria protein (*fimA*, *fimD*, *pliT*, and *hofP*), chemotaxis (*cheA*, *cheR*, and *mcp*), biofilm formation (*glpB*, *glpC*, *glpE*, *hmsH*, and *algD*), type II secretion system (*gspE*, *gspF*, *gspJ*, *gspK*, and *gspL*) were downregulated significantly in the *rpoS*-RNAi strain (Tables 1, 2), which suggested that *rpoS* was involved in stress response and virulence regulation of *E. cloacae*.

**Table 1:** Oligonucleotides used in producing shRNA for stable gene silencing.

Target gene	shRNA sequence for stable gene silence (5′-3′)
*rpoS*-shRNA-99	F: TGGAACCCAGTGATAACGATTTTTCAAGAGAAAATCGTTATC
	ACTGGGTTCCTTTTTTT
	R: GTACAAAAAAAGGAACCCAGTGATAACGATTTTCTCTTGAA
	AAATCGTTATCACTGGGTTCCATGCA
*rpoS*-shRNA-121	F: TGCTGAAGAAGAGCTGTTATCGTTCAAGAGACGATAACAGC
	TCTTCTTCAGCTTTTTTT
	R: ACGTACGACTTCTTCTCGACAATAGCAAGTTCTCTGCTATTGT
	CGAGAAGAAGTCGAAAAAAACATG
*rpoS*-shRNA-546	F: TGCGCGAGTTGTCCCATAAACTTTCAAGAGAAGTTTATGGGA
	CAACTCGCGCTTTTTTT
	R: GTACAAAAAAAGCGCGAGTTGTCCCATAAACTTCTCTTGAA
	AGTTTATGGGACAACTCGCGCATGCA
*rpoS*-shRNA-585	F: TGGAAGAGATCGCAGAGCAACTTTCAAGAGAAGTTGCTCTG
	CGATCTCTTCCTTTTTTT
	R: GTACAAAAAAAGGAAGAGATCGCAGAGCAACTTCTCTTGAA
	AGTTGCTCTGCGATCTCTTCCATGCA
*rpoS*-shRNA-750	F: TGCAGGACGATGACATGAAACATTCAAGAGATGTTTCATGTC
	ATCGTCCTGCTTTTTTT
	R: GTACAAAAAAAGCAGGACGATGACATGAAACATCTCTTGAA
	TGTTTCATGTCATCGTCCTGCATGCA

**Table 2:** The genes regulate by *rpoS* in *Enterobactor cloacae*.

Transcript ID	Gene	Gene function description	Fold change
BFV67_RS08030	*flgC*	Flagellar basal body rod protein FlgC	−4.81
BFV67_RS08045	*flgF*	Flagellar basal body rod protein FlgF	−3.73
BFV67_RS13720	*fliF*	Flagellar basal body M-ring protein FliF	−2.80
BFV67_RS13725	*fliG*	Flagellar motor switch protein FliG	−1.64
BFV67_RS13730	*fliH*	Flagellar assembly protein FliH	−2.51
BFV67_RS13735	*fliI*	Flagellum-specific ATP synthase FliI	−3.04
BFV67_RS13745	*fliK*	Flagellar hook length control protein FliK	−2.72
BFV67_RS13765	*fliO*	Flagellar type III secretion system protein FliO	−1.94
BFV67_RS13410	*flhA*	Flagellar biosynthesis protein FlhA	−3.42
BFV67_RS13415	*flhB*	Flagellar type III secretion system protein FlhB	−2.93
BFV67_RS13405	*flhE*	Flagellar protein FlhE	−2.78
BFV67_RS07480	*fimA*	Fimbrial protein FimA	−3.54
BFV67_RS02885	*fimD*	Fimbrial protein FimD	−4.30
BFV67_RS18255	*pliT*	Type IV pilus twitching motility protein PilT	−2.60
BFV67_RS20445	*hofP*	Pilus assembly protein HofP	−1.68
BFV67_RS13485	*cheA*	Chemotaxis protein CheA	−1.88
BFV67_RS13435	*cheR*	Protein-glutamate O-methyltransferase CheR	−1.90
BFV67_RS02310	*mcp*	Methyl-accepting chemotaxis protein	−3.26
BFV67_RS15215	*glpB*	Glycerol-3-phosphate dehydrogenase subunit GlpB	−1.75
BFV67_RS15220	*glpC*	Glycerol-3-phosphate dehydrogenase subunit GlpC	−2.17
BFV67_RS20590	*glpE*	Glycerol-3-phosphate dehydrogenase subunit GlpE	−3.80
BFV67_RS18390	*hmsH*	Biofilm formation protein HmsH	−3.35
BFV67_RS10685	*algD*	GDP-mannose pyrophosphatase	−2.51
BFV67_RS06885	*gspE*	Type II secretion system protein GspE	−1.62
BFV67_RS06895	*gspF*	Type II secretion system protein GspF	−2.70
BFV67_RS06915	*gspJ*	Type II secretion system protein GspJ	−3.51
BFV67_RS06920	*gspK*	General secretion pathway protein GspK	−3.36
BFV67_RS06925	*gspL*	Type II secretion system protein GspL	−2.84
BFV67_RS06945	*iagB*	Type III secretion system invasion protein IagB	−2.94
BFV67_RS00880	*ompA*	Outer membrane protein OmpA	−2.15
BFV67_RS10680	*luxR*	LuxR family transcriptional regulator	−1.52
BFV67_RS20195	*bfr*	Bacterioferritin	−2.01
BFV67_RS06485	*dps*	DNA starvation phase protection protein Dps	−2.55
BFV67_RS13875	*funC*	Fumarate hydratase	−2.16
BFV67_RS08635	*katE*	Catalase	−1.56
BFV67_RS20875	*uspB*	Universal stress protein UspB	−1.73
BFV67_RS13505	*uspC*	Universal stress protein UspC	−3.44
BFV67_RS09310	*uspE*	Universal stress protein UspE	−3.92
BFV67_RS09755	*uspF*	Universal stress protein UspF	−2.51

The functions of the 458 DEGs between the *rpoS*-RNAi and wild-type *E. cloacae* were analyzed by Go tools and categorized into different enriched functional groups, among which the important functions included localization, transport, establishment of localization, membrane, transporter activity, active transmembrane transporter activity and GTPase activity (Figure 2B). The 458 DEGs were also performed KEGG pathway enrichment analysis and enriched in 67 KEGG pathways, including two-component system, ABC transporters, flagellar assembly, bacterial chemotaxis, quorum sensing and so on (Figures 2B,C).

### Effect of *rpoS* on the survival of *Enterobacter cloacae* under environmental stresses

**Figure 3:**
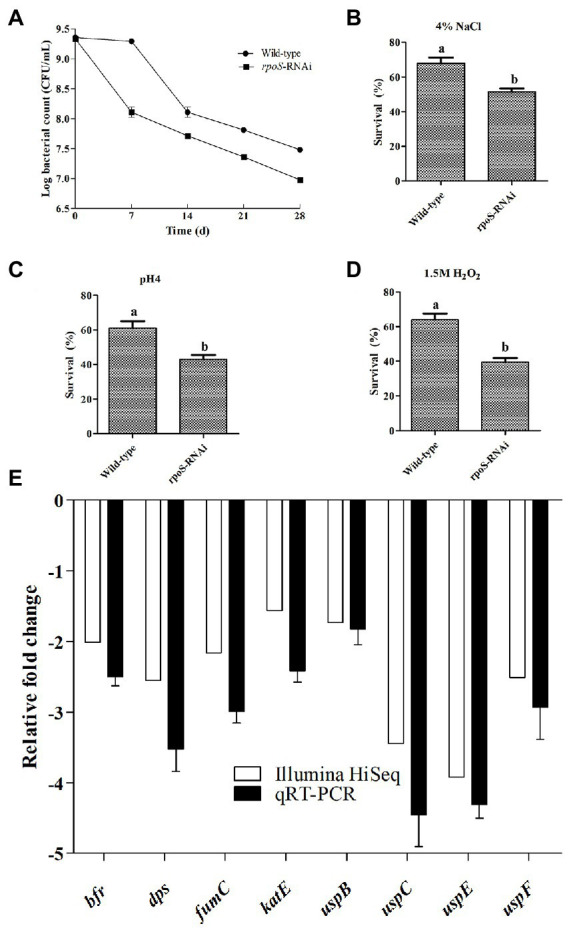
Effects of *rpoS* knockdown on stress resistance in *Enterobacter cloacae*. **(A)** Survival of wild-type and *rpoS*-RNAi strains under starvation. **(B)** Survival of wild-type and *rpoS*-RNAi strains in 4% NaCl. **(C)** Survival of wild-type and *rpoS*-RNAi strains in pH 4. **(D)** Survival of wild-type and *rpoS*-RNAi strains in 1.5 mmol/l H_2_O_2_. **(E)** Effects of *rpoS* on stress related genes expression in *E. cloacae*. The values marked with different letters (a, b) are significantly different (*p* < 0.05).

To investigate the effect of *rpoS* on the survival rate of *E. cloacae* under environmental stress conditions, the survival rates of the *rpoS*-RNAi and wild-type *E. cloacae* under environmental stress conditions (starvation, 4% NaCl, 1.5 mM H_2_O_2_ or pH 4) were measured. As shown in Figure 3, the survival rates of the *rpoS*-RNAi strain under these stress conditions decreased compared with the wild-type strain. After 28 d of starvation, the survival rate of the wild-type strain was 3.50-fold higher than the *rpoS*-RNAi strain (Figure 3A). Similarly, After1 h of exposure to 4% NaCl, 1.5 mM H_2_O_2_, and pH 4, survival rates of the WT were 1.32, 1.42 and 1.62-fold higher than the *rpoS*-RNAi strain, respectively (Figures 3B–D). The results of transcriptome analysis and qPCR verification also suggested that after the expression the expression of *rpoS* was inhibited, the resistance to environmental stress related genes including bacterioferritin (*bfr*), DNA starvation phase protection protein (*dps*), fumarate hydratase (*fumC*), catalase (*cat*), universal stress proteins (*uspB*, *uspC*, *uspE*, and *uspF*) were significantly down-regulated (Figure 3E).

### Effect of *rpoS* on biofilm formation ability of *Enterobacter cloacae*

**Figure 4:**
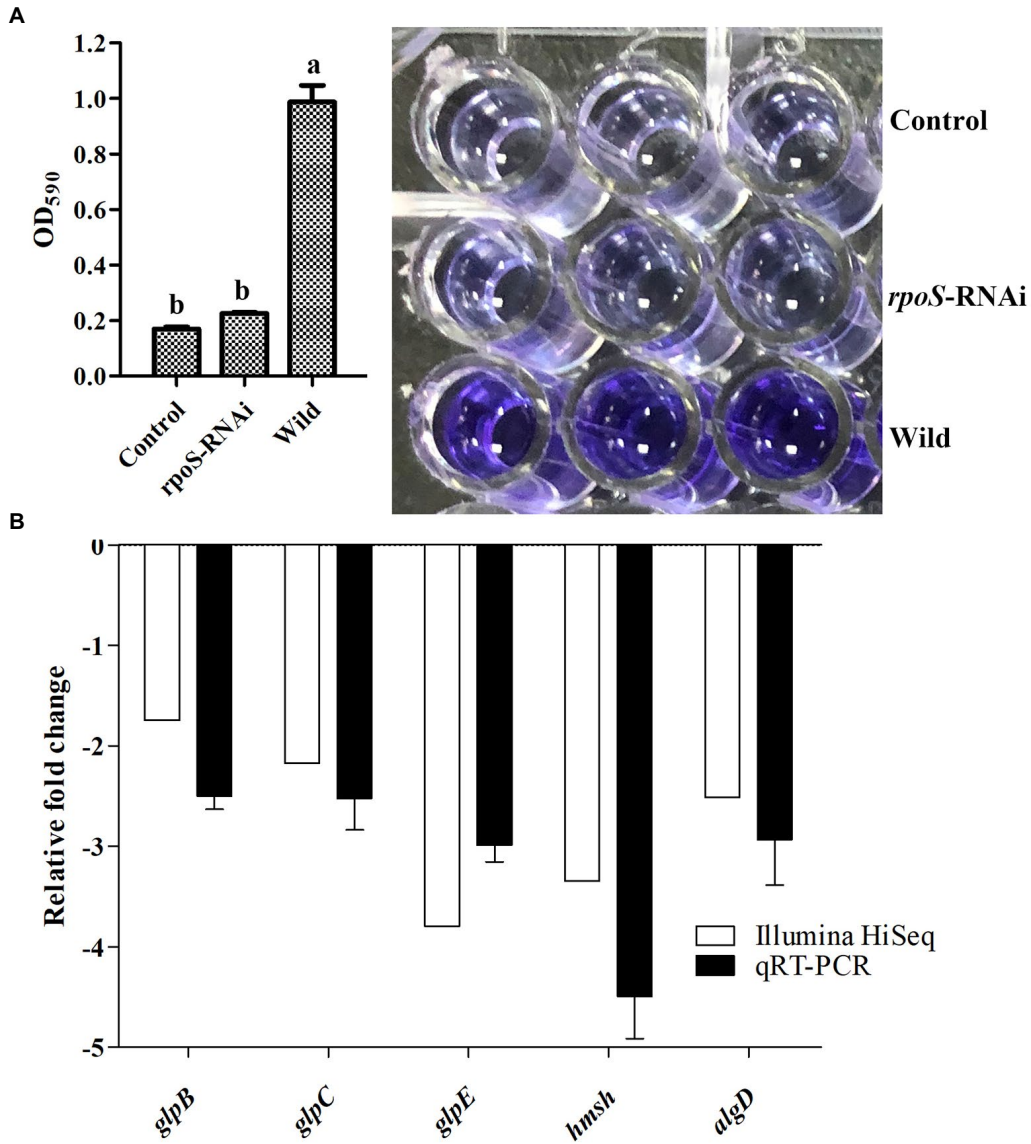
Effect of *rpoS* on biofilm formation in *E. cloacae*. **(A)** Biofilm formation ability of wild-type and *rpoS*-RNAi strains. **(B)** Expressions of biofilm formation related genes in *rpoS*-RNAi strain; The values marked with different letters (a, b) are significantly different (*p* < 0.05).

The biofilm formation ability compared between the *rpoS*-RNAi and wild-type strains was shown in Figure 4. The ability to form biofilms was decreased significantly in the *rpoS*-RNAi strain compared to the wild-type strain (Figure 4A). The results of transcriptome analysis and qPCR verification also showed the biofilm formation-related genes including *glpB*, *glpC*, *glpE*, *hmsH*, and *algD*, were significantly down-regulated (Figure 4B), which suggested that that *rpoS* positively regulated the expression of the biofilm formation-related genes.

### Effect of *rpoS* on motility of *Enterobacter cloacae*

**Figure 5:**
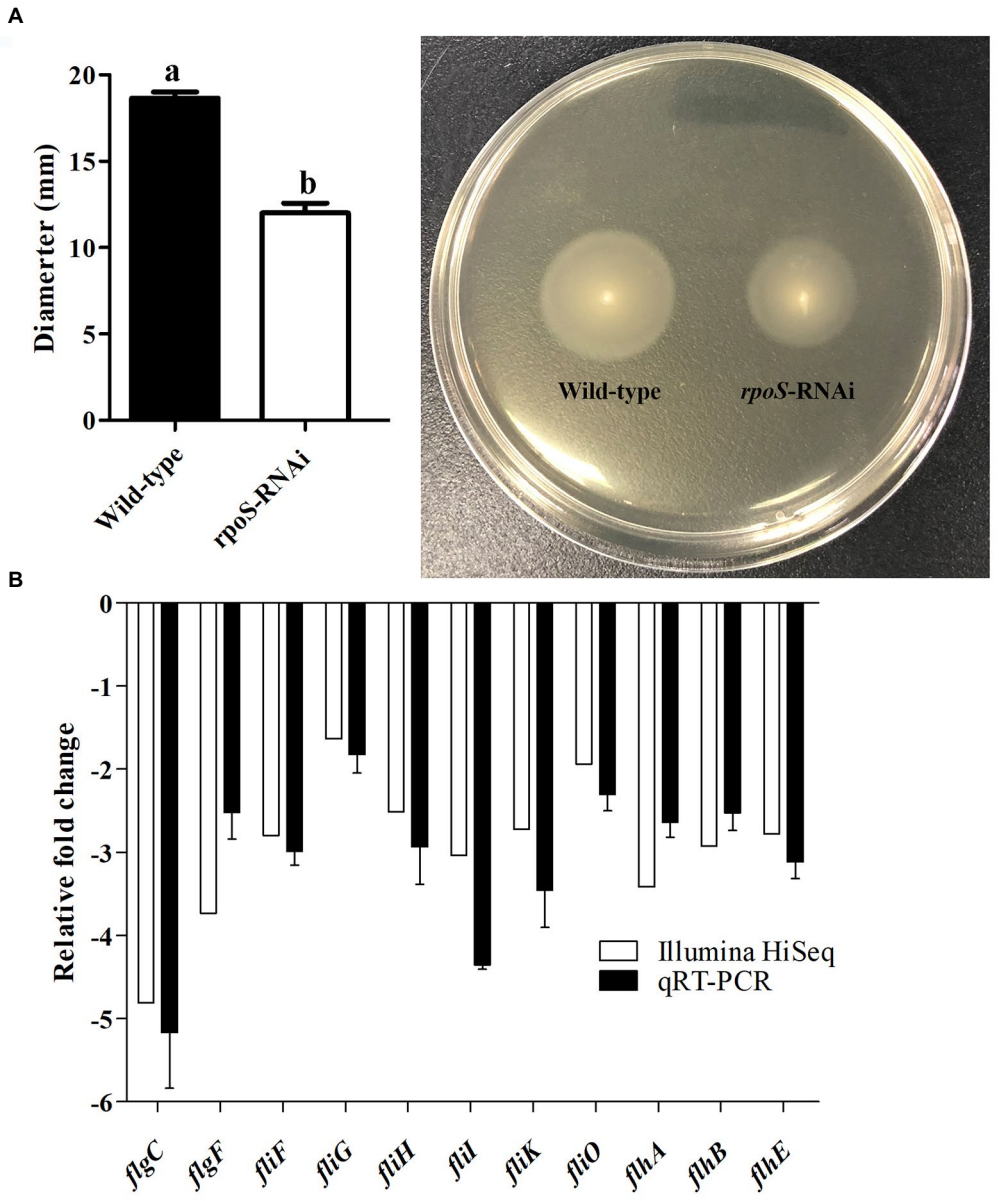
Effects of *rpoS* on motility of *Enterobacter cloacae*. **(A)** Detection of motility of *E. cloacae* wild-type and *rpoS*-RNAi strains; **(B)** Expressions of motility-related genes in *rpoS*-RNAi strain; The values marked with different letters (a, b) are significantly different (*p* < 0.05).

The effect of *rpoS* expression on the motility of *E. cloacae* are shown in Figure 5. The *rpoS*-RNAi strain was motile, but the diameters of the colonies (12.0 ± 0.8 mm) after 24 h of culture were significantly smaller than that of the wild-type strain (18.7 ± 0.5 mm) (Figure 5A). In addition, the genes related to flagellar assembly (*flgC*, *flgF*, *fliF*, *fliG*, *fliH*, *fliI*, *fliK*, *fliO*, *flhA*, *flhB*, and *fimC*) were significantly down-regulated (Figure 5B). These results confirmed that the reduction in *E. cloacae* motility may be due to knockdown of *rpoS*.

### Knockdown of *rpoS* reduced adhesion ability of *Enterobacter cloacae*

**Figure 6:**
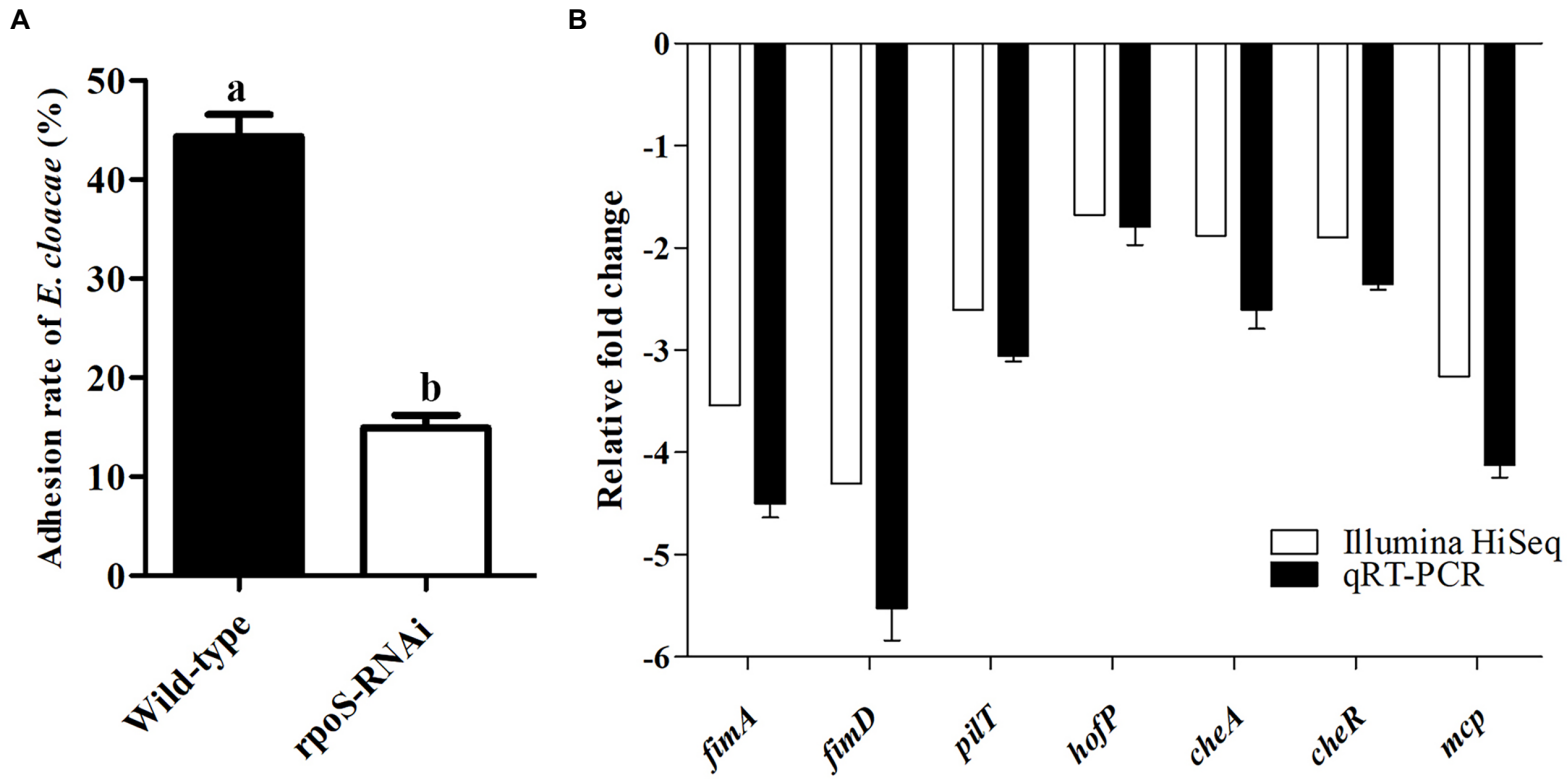
Effects of *rpoS* on adhesion of *Enterobacter cloacae*. **(A)** The adhesion rate of *E. cloacae* wild-type and *rpoS*-RNAi strains to 293 T cells; **(B)** Expressions of adhesion-related genes in *rpoS*-RNAi strain; The values marked with different letters (a, b) are significantly different (*p* < 0.05).

Prior to studying the effects of *rpoS* on adhesion ability of *E. cloacae*, we tested the adhesion ability of the *rpoS*-RNAi and wild-type *E. cloacae* to live 293 T cells. The adhesion rate of the *rpoS*-RNAi strain into 293 T cells was 14.95%, while the adhesion rate of the wild-type strain into 293 T cells was 44.35% (Figure 6A). In addition, the results of transcriptome analysis and qPCR verification also showed some genes related to pilus biogenesis (*fimA*, *fimD*, *pliT*, *hofP*), bacterial chemotaxis (*cheA*, *cheR*, *mcp*) were significantly down-regulated after the expression inhibition of *rpoS* (Figure 6B). These results suggested that *rpoS* involved in the process of *E. cloacae* adhesion.

### Effect of *rpoS* on virulence of *Enterobacter cloacae*

**Figure 7:**
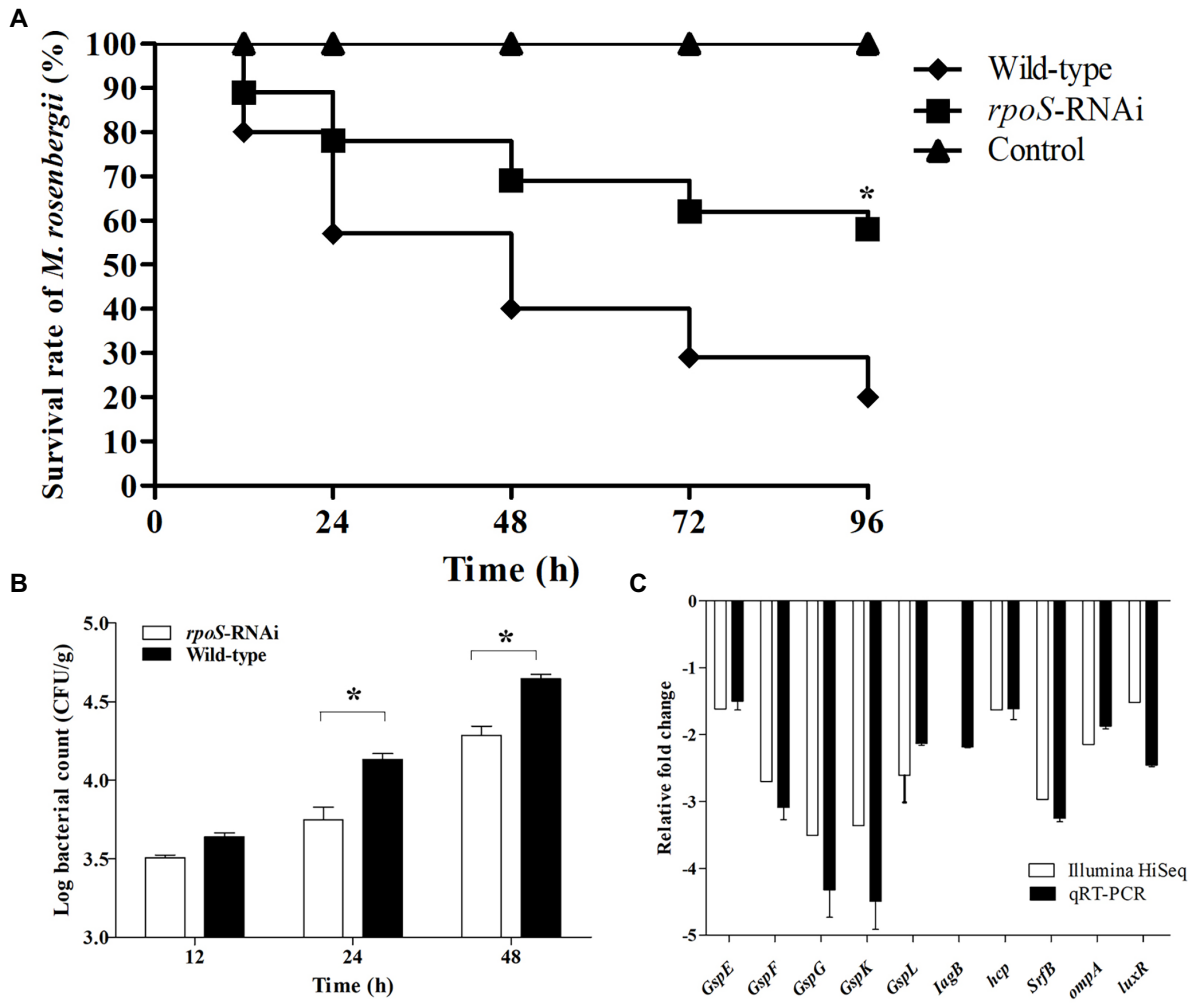
Effects of *rpoS* on virulence of *Enterobacter cloacae.* **(A)** Survival of *M. rosenbergii* after infected with *E. cloacae* wild-type and *rpoS*-RNAi strains; **(B)** Bacterial burden of *M. rosenbergii* after infected with *E. cloacae* wild-type and *rpoS*-RNAi strains; **(C)** Expressions of virulence-related genes in *rpoS*-RNAi strain; The asterisk indicated significant differences between the *E. cloacae* wild-type and *rpoS*-RNAi infected groups (*p* < 0.05).

Comparison of pathogenicity between the *rpoS*-RNAi and wild-type *E. cloacae* were showed in Figure 7. The virulence test showed the *M. rosenbergii* injected with the wild-type *E. cloacae* showed 58% survival rate at 96 hpi, while the *M. rosenbergii* injected with the *rpoS*-RNAi *E. cloacae* showed 20% survival rate (Figure 7A). Besides, the bacterial burden of the *rpoS*-RNAi *E. cloacae* in *M. rosenbergii* (4.29 Log10 CFU/g) was significantly lower than that of the wild-type *E. cloacae* (4.65 Log10 CFU/g) at 48 hpi (Figure 7B). Additionally, the expression of multiple virulence genes, such as *gspE*, *gspF*, *gspJ*, *gspK*, *gspL*, *iagB*, *luxR*, and *ompA* were significantly down-regulated in the *rpoS*-RNAi strain (Figures 7B,C). These results suggested that *rpoS* was likely involved in the regulation of bacterial virulence.

## Discussion

It is well known that aquatic pathogenic bacteria are frequently subjected to a variety of stresses in the aquatic environments or host systems, and must adapt to survive in these environmental stresses, especially during infection (Sun et al., 2016; Awan et al., 2018; Gao et al., 2018, 2022). As a result, to investigate the environmental adaptation mechanism of bacterial pathogens, many studies focused to identify the key stress resistance related genes (Kamareddine et al., 2018; Zhan et al., 2021). Among these, RpoS has been characterized as an alternative sigma factor responsible for the regulation of stress response genes in many bacteria (Guo et al., 2019; Fernández-Gómez et al., 2020). The *rpoS* gene encodes RpoS, and has been proved to be critical for adaptation to environment stresses many bacteria, but limited information is available regarding the role of *rpoS* in *E. cloacae*. To address this issue, the phenotypes and transcriptome of wild-type and *rpoS*-RNAi strains were compared to clarify the role of *rpoS* in the *E. cloacae*.

In this study, the transcriptome analysis generated a novel dataset describing the functional response of *E. cloacae* after knockdown of *rpoS*. Our results suggested that the *rpoS* may regulate the expression of at least 488 genes, including 458 downregulated genes and 30 upregulated genes. These DEGs were mainly were involved into stress resistance, flagellar assembly, bacterial chemotaxis, biofilm formation, two-component system, quorum sensing, adherence, and secretion, etc., which suggested that RpoS is a critical regulator of stress resistance and virulence in *E. cloacae*. The RpoS can positively regulated a lot of stress resistance related genes in our study as previously reported in other bacterial, e.g., Weber et al. (2005) reported that RpoS controls the expression of approximately 10–23% of all genes in *E. coli*, and these genes were involved in responses to involved in responses to varying environmental conditions. Meanwhile, RpoS has been also shown to regulate the expression of many virulence genes in several pathogens (Chang and Lee, 2018; Yin et al., 2018). For example, RpoS could activate the expression of the plasmid-borne *spvR* and *spvABCD* related virulence in *Salmonella typhimurium* (Waterman and Small, 2003). Additionally, the analysis of KEGG enrichment may help us to understand the molecular regulation mechanism of *rpoS* in *E. cloacae*. KEGG analysis in our study showed that the DEGs of the *rpoS*-RNAi and wild-type strain were mainly enriched in two-component system, ABC transporters, flagellar assembly, bacterial chemotaxis and quorum sensing KEGG pathways, which associated with bacterial survival and virulence (Higgins, 2001; Joelsson et al., 2007; Reading et al., 2009; Wang et al., 2015). These results indicated that *rpoS* was involved in stress response and virulence regulation of *E. cloacae*.

To further determine the functional roles of *rpoS* in survival of *E. cloacae*, the effects of *rpoS* on survival under stress conditions and the biofilm formation were investigated. In this study, we found that in *E. cloacae*, knockdown of *rpoS* led to decreased survival rates after starvation, acid, osmotic, and oxidative stresses. Similar results were also observed in *Yersinia pseudotuberculosis*, *rpoS* mutation led to decreased survival rates after oxidative, acid, osmotic and 42°C heat shock stresses (Guan et al., 2015). Zhan et al. (2021) also reported that membrane formation, acid tolerance and drying tolerance ability was decreased significantly in the mutant strain Δ*rpoS* of *Cronobacter sakazakii*. As expected, knockdown of *rpoS* in *E. cloacae* also resulted in decreased expression of the majority of RpoS-dependent stress resistance related genes (*bfr*, *dps*, *funC*, *katE*、*uspB*, *uspC*, *uspE*, *uspF*). Furthermore, the previous studies have showed that *rpoS* is involved in biofilm production in many bacteria, and biofilm formation could enhance the resistance to natural environment stresses and host immune killing (Kulikalova et al., 2014; Beshiru and Igbinosa, 2018; Olwal et al., 2018). Our studies also showed that knockdown of *rpoS* could decrease the biofilm production in *E. cloacae*, which demonstrated tha*t rpoS* plays an important role in *E. cloacae* biofilm production. Additionally, this study suggested that *rpoS* can positively regulate the expression of biofilm formation related genes, such as *glpB*, *glpC*, *glpE*, *hmsH*, and *algD*. The results of the present study indicated that *rpoS* has an important effect on the regulation of stress response genes in *E. cloacae*, and thus allows the bacteria to survive in adverse environments.

In addition to regulating environmental resistance functions, RpoS is essential for virulence regulation in several pathogens (Fang et al., 1992; Dong and Schellhorn, 2010; Huang et al., 2019). In this study, the effects of *rpoS* on *E. cloacae* virulence were evaluated by motility test, adhesion test and virulence test. It is well known that motility is important for colonization, adhesion and causing disease of bacteria. Our studies showed that RNAi-treated bacteria exhibited significantly impaired abilities of motility, consistent with the findings in *V. vulnificus*, *P. aeruginosa*, and *Y. pseudotuberculosis* (Dong and Schellhorn, 2010; Guan et al., 2015; Huang et al., 2019). Meanwhile, we demonstrated that *rpoS* can positively regulate the flagellum expression to control motility in *E. cloacae*, consistent with previous findings that *rpoS* is necessary for motility (Wang et al., 2012). Furthermore, adhesion of pathogenic bacteria to the mucus of host animals is often the initial step of infection, and *rpoS* have been proved to regulate adhesion in other bacteria (Adnan et al., 2017). In the present study, the knockdown of *rpoS* can significantly reduce the adhesion, consistent with that *rpoS* can positively regulate adhesion in other bacteria (Huang et al., 2019). Finally, the virulence test showed that the *rpoS*-RNAi *E. cloacae* exhibited attenuated pathogenicity and colonization to *M. rosenbergii*. Similar results were also observed in *Vibrio anguillarum*, σ^S^ mutation led to reduced production of hemolysin, catalase and phospholipase. *rpoS* mutation led to was reduced production of hemolysin, catalase and phospholipase, as well as decreased virulence to zebra fish (Ma et al., 2009). These findings revealed that *rpoS* was involved in the regulation of *E. cloacae* virulence.

In conclusion, these findings suggest the *rpoS* gene not only contributes survival under adverse environments, but also is crucial for the virulence regulation in *E. cloacae*. The results will provide a better understanding in environmental adaptation and pathogenesis mechanism of *E. cloacae*, and provides reference for the prevention of *E. cloacae* caused diseases in aquaculture.

## Data availability statement

The datasets presented in this study can be found in online repositories. The names of the repository/repositories and accession number(s) can be found in NCBI with BioProject ID PRJNA857757.

## Ethics statement

All treatments of prawns in this study were strictly in accordance with the guidelines of Animal Experiment Ethics Committee of Yangzhou University. The protocol was approved by Animal Experiment Ethics Committee of Yangzhou University.

## Author contributions

XG conceived, designed, and carried out the experiments, analyzed the data, and wrote the manuscript. QQ, YZ, ZC, JX, WX, QJ, and JW participated in performing experiments, and analyzing the data. XZ participated in designing the experiments and revising the manuscript. All authors contributed to the article and approved the submitted version.

## Funding

This work was supported by the National Natural Science Fund (Nos. 31972830 and 32202982), Natural Science Foundation of Jiangsu Province (no. BK20220584), earmarked fund for Jiangsu Agricultural Industry Technology System (JATS [2022] 501, JATS [2022] 504), “JBGS” Project of Seed Industry Revitalization in Jiangsu Province (JBGS [2021] 120), Project of Major Agricultural Technology Cooperative Promotion in Jiangsu Province (no. 2021-ZYXT-05-4).

## Conflict of interest

The authors declare that the research was conducted in the absence of any commercial or financial relationships that could be construed as a potential conflict of interest.

## Publisher’s note

All claims expressed in this article are solely those of the authors and do not necessarily represent those of their affiliated organizations, or those of the publisher, the editors and the reviewers. Any product that may be evaluated in this article, or claim that may be made by its manufacturer, is not guaranteed or endorsed by the publisher.
