# Supplementary material for: Transcriptomic and phenotype analysis revealed the role of rpoS in stress resistance and virulence of pathogenic Enterobacter cloacae from Macrobrachium rosenbergii
Source: Front Microbiol. 2022 Nov 10;13:1030955. doi: 10.3389/fmicb.2022.1030955 (PMC9684176; doi:10.3389/fmicb.2022.1030955)
Supplement: Supplementary file 1 [file Table_1.DOCX]

**TABLE S1** qRT-PCR primers used in this study.

| Target gene | PCR primers sequence (5′-3′) | Transcript ID |
| --- | --- | --- |
| 16S rRNA | CTAATACCGCATAATGTCGC | GenBank MF197498.1 |
|  | TGTGGCTGGTCATCCTCT |  |
| *rpoS* | TGAGAACGGAGCAGAGGC | GenBank Y13230.1 |
|  | GCGAGGCAACATCACCAC |  |
| *bfr* | GCAATGACGGCTATGGCTTAC | BFV67_RS20195 |
|  | CAAATCCTGACGCCACAGTAAA |  |
| *dps* | ATTCGGTATCAGCCAGTTCAG | BFV67_RS06485 |
|  | AATAAGGAAGGTTTAGGCATCG |  |
| *funC* | GACGAAGTCTTTATCCTCCCG | BFV67_RS13875 |
|  | AACACTTTCCACAGTTCCACC |  |
| *katE* | GTGCCTGCCCTGGCGATGTT | BFV67_RS08635 |
|  | AGCAAGCCTGCGGCGAAA |  |
| *uspB* | ACCGTAATCAGGGTGACAAA | BFV67_RS20875 |
|  | AAGGCTGCGTGACCGTAC |  |
| *uspC* | GAAAGGAGCCATCTCACCC | BFV67_RS13505 |
|  | CGACGGATTCACTAAAGCA |  |
| *uspE* | GACGCTGGATGTGGTAGGG | BFV67_RS09310 |
|  | AATGACCAGTGCGGAAGC |  |
| *uspF* | CTGGATATGTGGGTTATGAAGA | BFV67_RS09755 |
|  | CAGCACCTGAAGCAGAATGT |  |
| *glpB* | GCATTCGCCTGCATACCC | BFV67_RS15215 |
|  | CGTCCAGATTTCGCTCACC |  |
| *glpC* | CTATGTGAATTACAACCACCCG | BFV67_RS15220 |
|  | GTAAACCCGTTAGCAATCAGC |  |
| *glpE* | AAAAGCGGTGCTGGTGGA | BFV67_RS20590 |
|  | GCTGCTGTTGCCGTGGTAG |  |
| *hmsH* | ACCATGAGATGTATCGCCATTA | BFV67_RS18390 |
|  | GTTGTCCGTAACCCAGCAGA |  |
| *algD* | TGAAAGTCGCATCAAAGC | BFV67_RS10685 |
|  | GGCTGATACTCGGCAAGA |  |
| *flgC* | CGGACAGACTGGTGTATGAGC | BFV67_RS08030 |
|  | TCGACGTTGGCCTGGTAG |  |
| *flgF* | GTAACGGGAACATCCAGGTGA | BFV67_RS08045 |
|  | AGGGTTGAGCGCCGAAAT |  |
| *fliF* | ATTCGGTATCAGCCAGTTCAG | BFV67_RS13720 |
|  | AATAAGGAAGGTTTAGGCATCG |  |
| *fliG* | CGTCAACGCCAACGACTA | BFV67_RS13725 |
|  | CACTCTGCGGTTCCATAAA |  |
| *fliH* | GACGCCCGTGGTGGATAA | BFV67_RS13730 |
|  | TGCAACCGCCGTGATGTAA |  |
| *fliI* | TGCTGTCTCGTCGTCTGGC | BFV67_RS13735 |
|  | GGGCGTAGTGTTTCTCGGTTAT |  |
| *fliK* | CGTCGCTTGATACGCTGGTA | BFV67_RS13745 |
|  | GCTCGTGAGGTGCTGGACT |  |
| *fliO* | GTCACGGCGTCAAACATCAG | BFV67_RS13765 |
|  | CAAACTCTTCATCACGGACTGG |  |
| *flhA* | CGGTATCGTGGTGTTCGTCA | BFV67_RS13410 |
|  | TCCCGTCCAGCACAAAGC |  |
| *flhB* | TGAATCTGGTCGGGCTCTG | BFV67_RS13415 |
|  | GTGCGGGTCACCTTCCAT |  |
| *flhE* | TGGCAGGCGAGCAGCATA | BFV67_RS13405 |
|  | AGTTCCAGACCACCAGACCC |  |
| *fimA* | GGTGCGTTATGTCGGGTCG | BFV67_RS07480 |
|  | TTTAGTGAGGCTATCGGTGTTC |  |
| *fimD* | CGACAGCGTTTGGGTAAGC | BFV67_RS0288 |
|  | GACCAGGAGGCATTGAGGTAG |  |
| *pliT* | CCGGGTTGATTCTGGTGAC | BFV67_RS18255 |
|  | CAACGCTCGCTCTGATAGATAA |  |
| *hofP* | ATAATGCTGTGGCTGCTGG | BFV67_RS20445 |
|  | CAGATTACGGTTCTCCTGTGC |  |
| *cheA* | GACGAAGTCTTTATCCTCCCG | BFV67_RS13485 |
|  | AACACTTTCCACAGTTCCACC |  |
| *cheR* | CCAGAACAGCGCAGAGTGG | BFV67_RS13435 |
|  | AGCGTATCGGCAAGGGTG |  |
| *mcp* | TTACTGGCCGTTATGACCCT | BFV67_RS02310 |
|  | TGCACCATCTGCTCACCCT |  |
| *gspE* | GGTGGCCGATGACGAAAC | BFV67_RS06885 |
|  | CGCTGGCATACTTGAGATAGAA |  |
| *gspF* | GCGGTGAGCGATTTCCTG | BFV67_RS06895 |
|  | ATAGCGGGCGCTGTTGAT |  |
| *gspJ* | TGCCTGGAGCCTGATGGA | BFV67_RS06915 |
|  | CGCGATACAGACGCCCTTC |  |
| *gspK* | GGCTGGGAAAGTATGGACG | BFV67_RS06920 |
|  | ATCGGTATTGCCGGTGTAG |  |
| *gspL* | GCGGCGAAAGGGATGAAG | BFV67_RS06925 |
|  | TGATGACGCATACTCGGAAA |  |
| *iagB* | GTAGGCTGGAACCACGAC | BFV67_RS06945 |
|  | TGGAGGTACAGGGCTCAT |  |
| *ompA* | GGCGATGGTGCTGAAAGAG | BFV67_RS00880 |
|  | TGTTGCTGGCGATAGGGTT |  |
| *luxR* | TGACCCTGTTATCATTCG | BFV67_RS10680 |
|  | TCATACTGGACACTTTCG |  |
