# Supplementary material for: Transcriptomic and phenotype analysis revealed the role of rpoS in stress resistance and virulence of pathogenic Enterobacter cloacae from Macrobrachium rosenbergii
Source: Front Microbiol. 2022 Nov 10;13:1030955. doi: 10.3389/fmicb.2022.1030955 (PMC9684176; doi:10.3389/fmicb.2022.1030955)
Supplement: Supplementary file 2 [file Table_2.DOCX]

**Table S2 Quality evaluation of transcriptome sequencing data**

| Sample name | *rpoS*-RNAi-1 | *rpoS*-RNAi-2 | *rpoS*-RNAi-3 | XL3-1-1 | XL3-1-2 | XL3-1-3 |
| --- | --- | --- | --- | --- | --- | --- |
| Raw reads | 11797418 | 11000270 | 11036078 | 11908214 | 9999666 | 15104910 |
| Clean reads | 11583012 | 10789068 | 10939872 | 11649402 | 9878616 | 14998822 |
| Q20(%) | 97.95 | 97.88 | 98.78 | 98.02 | 97.55 | 97.89 |
| Q30(%) | 93.94 | 93.81 | 95.97 | 94.1 | 93.07 | 93.79 |
| Total  mapped | 10036777 (86.65%) | 8666885 (80.33%) | 9620252 (87.94%) | 11354715 (97.47%) | 9586042 (97.04%) | 14468246 (96.46%) |
| Multiple mapped | 313470  (2.71%) | 268641 (2.49%) | 163530 (1.49%) | 286395  (2.46%) | 498094 (5.04%) | 951137  (6.34%) |
| Uniquely mapped | 9723307 (83.94%) | 8398244 (77.84%) | 9456722 (86.44%) | 11068320 (95.01%) | 9087948 (92%) | 13517109 (90.12%) |
